# The nonsense-mediated decay RNA-surveillance pathway facilitates the hypoxia response by *C. elegans*

**DOI:** 10.1126/sciadv.aej4259

**Published:** 2026-10-28

**Authors:** Calista Diehl, H. Robert Horvitz

**Affiliations:** Howard Hughes Medical Institute, Department of Biology, Massachusetts Institute of Technology Cambridge, MA 02139, USA.

## Abstract

Hypoxia (decreased O_2_) induces physiological, metabolic, and behavioral changes. The major hypoxia response pathway is conserved from nematodes to mammals and is driven by activation of the HIF (hypoxia inducible factor) family of transcription factors. Despite a fundamental role of the HIF pathway in regulating cellular homeostasis in response to hypoxic stress, interactions between the HIF pathway and cellular quality control pathways are largely unknown. Using an unbiased genetic screen, we found that in *C. elegans* nonsense-mediated decay (NMD), an evolutionarily conserved mechanism of RNA surveillance, acts in parallel to HIF-1 to selectively enhance specific aspects of the response to HIF-1 activation, possibly through coregulation of a distinct subset of HIF-1–up-regulated genes. Our findings reveal a functional integration between oxygen sensing and RNA surveillance and establish NMD as a key regulator of specific aspects of the HIF-1–driven transcriptional program and physiological response.

## INTRODUCTION

To survive, aerobic organisms must respond appropriately to changes in oxygen levels produced by different environments and physiological conditions. Low levels of O_2_ can induce hypoxia, which drives coordinated changes at the cellular, tissue, and organismal levels. In the nematode *Caenorhabditis elegans*, the hypoxia response is regulated by the evolutionarily conserved prolyl hydroxylase EGL-9 (EGLN, PHD, or HIF-PH in mammals) and the transcription factor HIF-1 [hypoxia-inducible factor; HIF-1α, HIF-2α (also known as EPAS1), and HIF-3α in mammals] (*1*–*3*). In normoxic conditions, EGL-9 uses ambient O_2_ to hydroxylate HIF-1, which is then polyubiquitinated and degraded; by contrast, in hypoxic conditions, EGL-9 lacks its O_2_ substrate and cannot hydroxylate HIF-1, leading to HIF-1 stabilization and activation, which drives adaption to hypoxia. In mammals, HIF induces critical transcriptional changes affecting cellular metabolism, mitochondrial morphology, angiogenesis, and erythropoiesis (*4*, *5*), making EGLN and HIF crucial for adaptation to hypoxic stress and hypoxia-driven aspects of embryonic development, such as blood vessel formation. EGLN is being explored as a potential therapeutic target for a wide range of disorders, including ischemic stroke, neurodegenerative diseases, and cancer (*6*–*8*). While the core hypoxia response pathway is well defined, if and how additional cellular pathways modulate HIF output to tune the hypoxia response remains poorly understood.

One well-studied mechanism for modulating transcriptional output involves RNA quality control pathways that regulate transcript stability and integrity. Nonsense-mediated decay (NMD) is an evolutionarily conserved RNA surveillance mechanism that identifies and degrades transcripts with premature termination codons (*9*). NMD plays an important role in maintaining cellular homeostasis (*10*–*13*), and it is estimated that 1 to 10% of all cellular transcripts in eukaryotes are either directly or indirectly NMD-regulated (*13*–*15*).

Similar to many other environmental challenges, hypoxia induces critical shifts in transcription, splicing, metabolism, and general cellular homeostasis. Nonetheless, interactions between the EGL-9/HIF-1 pathway and cellular quality control pathways, such as NMD, are largely unknown. Here we report the discovery that in *C. elegans*, the process of NMD is necessary for a complete hypoxia response. On the basis of the results of a genetic suppressor screen and behavioral and transcriptomic analyses, we conclude that NMD is required for specific HIF-1–dependent behavioral and stress-resistance responses by *C. elegans*. The effects of NMD on the hypoxia response are neither caused by a general down-regulation of HIF-1 activity nor through HIF-1–dependent regulation of NMD; instead NMD likely acts in parallel to HIF-1 to coregulate specific aspects of the hypoxia response. Our findings uncover an unexpected integration of oxygen-sensing and RNA-surveillance pathways in *C. elegans* and demonstrate that transcript quality control mechanisms can actively shape organismal adaptation to hypoxia.

## RESULTS

### *smg-1* function is necessary for complete HIF-1–driven down-regulation of egg laying

The activation of HIF-1 in *C. elegans* through either exposure to hypoxia or the loss of EGL-9 function results in numerous physiological changes. For example, HIF-1 activation promotes the retention of eggs inside the uterus, presumably to prevent the deposition of eggs in an unfavorable environment with limited oxygen (*16*, *17*). In e*gl-9(lf)* mutants HIF-1 is constitutively activated, and *egl-9(lf)* mutants retrain eggs and display the Egl (egg-laying abnormal) phenotype, even in normoxic (room air, ∼20% O_2_) conditions. Animals with this Egl phenotype have a uterus visibly bloated with an abnormally large number of developing eggs (fig. S1, A and B) and lay eggs at later embryonic stages than wild-type animals because eggs have spent longer in the uterus before being laid (Fig. 1, A to C). This Egl phenotype is completely dependent on a functional *hif-1(+)* gene, as eggs are not retained in *egl-9(−) hif-1(−)* double mutants (Fig. 1D and fig. S1C).

**Fig. 1.**
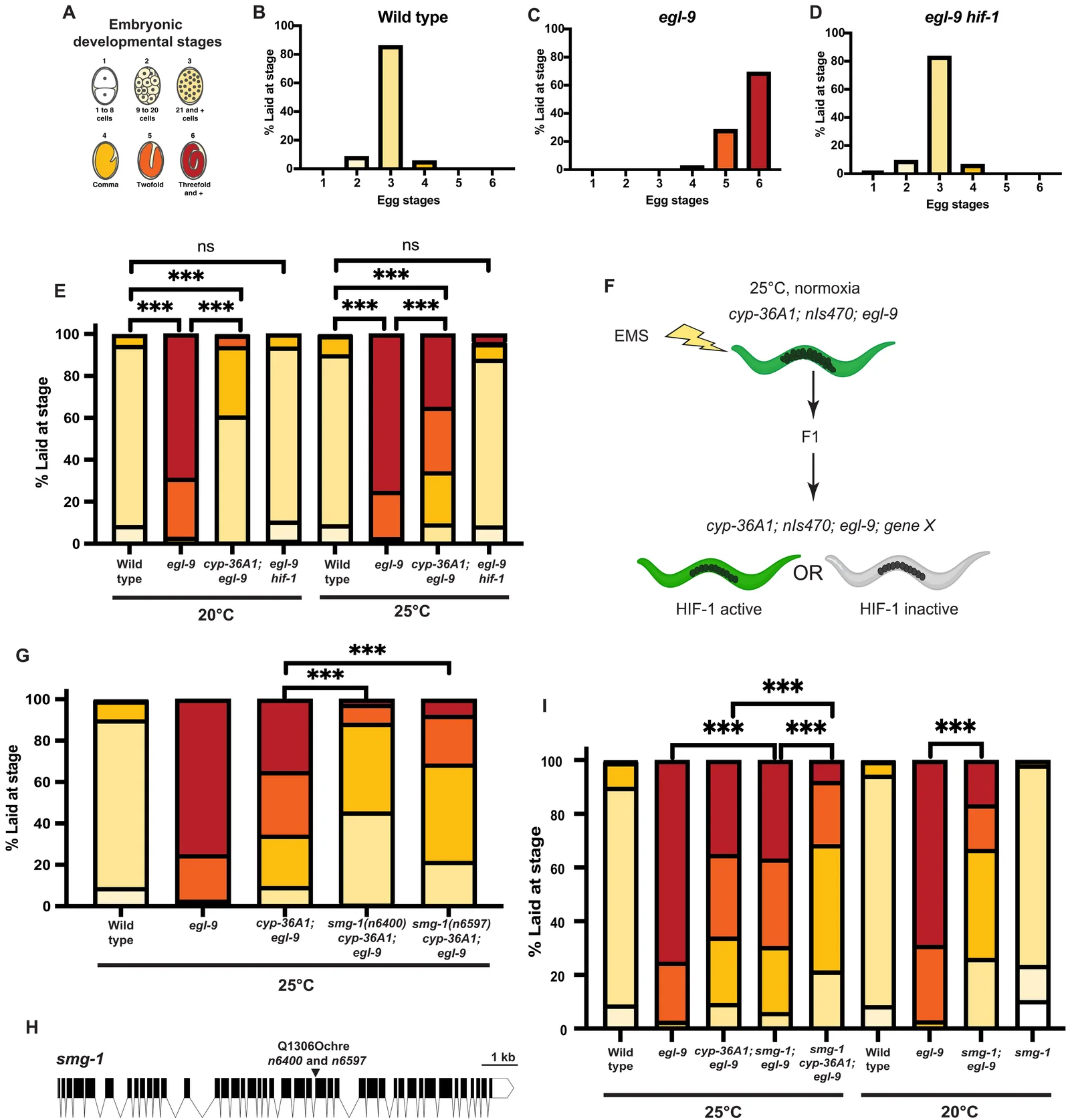
A *smg-1(lf)* mutation suppresses the *egl-9(lf)* Egl phenotype. (**A**) The six embryonic stages used to categorize recently laid eggs. Sections of bar graphs are matched by color. Figure adapted from Pender and Horvitz (*18*) under CC BY 4.0 (http://creativecommons.org/licenses/by/4.0) (**B**) Wild-type animals lay primarily stage 3 embryos. (**C**) *egl-9(lf)* mutants lay later stage embryos, producing an Egl phenotype. (**D**) *egl-9(lf) hif-1(lf)* has wild-type egg laying. (**E**) *egl-9* Egl phenotype is suppressed by a *cyp-36A1* null mutation at 20°C but much less so at 25°C. (**F**) EMS mutagenesis screen to isolate novel *egl-9* suppressors. Strains contain loss-of-function mutations in *egl-9* and the partial suppressor *cyp-36A1. nIs470*, a transcriptional reporter of the HIF-1 target *cysl-2*, is used to indicate HIF-1 activity. Created in BioRender. I. Kesisova (2026; https://BioRender.com/8jgtqyf). (**G**) Nonsense mutation *n6400* isolated in our screen suppresses *egl-9*. The identical point mutation in *smg-1* generated using CRISPR-Cas9 (*n6597* allele) similarly suppresses *egl-9*. (**H**) Schematic showing *smg-1* gene structure and the *n6400* and *n6597* Q1306Ochre point mutation in *smg-1*. (**I**) *smg-1* and *cyp-36A1* function additively. *smg-1(n6597)* suppresses *egl-9* at both 20° and 25°C. For all samples, *n* > 100 embryos.

Previously we showed that the cytochrome P450 gene *cyp-36A1* functions downstream of *hif-1* to mediate multiple aspects of the hypoxia response, including egg retention, through the production of a secreted hormone ligand that regulates a nuclear hormone receptor (*18*). In this study, we confirmed that loss of *cyp-36A1* function suppresses the Egl phenotype of *egl-9*, as *cyp-36A1; egl-9* double mutants laid earlier stage embryos than *egl-9* single mutants in normoxia. However, we further observed that this suppression was incomplete, particularly when animals were grown at the higher temperature of 25°C instead of the more typical 20°C (Fig. 1E). As the *cyp-36A1(gk824636)* allele that was used introduces a nonsense codon in the first quarter of the CYP-36A1 protein, it is unlikely that any functional CYP-36A1 protein is produced at any temperature, suggesting that the temperature dependence of the *cyp-36A1* suppression is not a consequence of CYP-36A1 protein thermolability. The incomplete suppression by *cyp-36A1* mutation of the Egl phenotype of *egl-9* mutants at 25°C led us to hypothesize that one or more additional genes function with HIF-1 to regulate egg laying independently of *cyp-36A1* activity.

To seek genes that, similar to *hif-1* and *cyp-36A1*, promote the hypoxia response, we performed an ethyl methanesulfonate (EMS) mutagenesis screen for suppressors of *egl-9*. Since a *cyp-36A1* mutation only partially suppressed the *egl-9* Egl phenotype at 25°C, we screened at 25°C (in normoxic conditions) for mutations that acted additively with a *cyp-36A1* mutation to further suppress the *egl-9* Egl phenotype to a wild-type non-Egl phenotype (Fig. 1F). To distinguish those mutations that decreased HIF-1 activity—i.e., either were in *hif-1* itself or that affected factors that acted upstream of HIF-1—we included in our mutagenized parental strain *nIs470*, a green fluorescent protein (GFP) transcriptional reporter for the gene *cysl-2*, which is expressed in response to HIF-1 activation (*19*, *20*). We sought non-Egl animals in which *nIs470* was expressed, indicating that while the downstream egg-laying behavior was affected, HIF-1 activity remained intact.

This screen generated an ochre nonsense mutation, *n6400*, in the gene *smg-1* (Fig. 1, G and H), which encodes the kinase that activates the process of NMD (*21*). Using CRISPR-Cas9, we remade this mutation to generate the *smg-1* allele *n6597* in our starting genetic background and observed a phenotype similar to that of our original isolate, indicating that the nonsense mutation in *smg-1* was the causative mutation (Fig. 1G). Because this *smg-1* allele is a nonsense mutation and causes a recessive phenotype, it likely causes a loss of *smg-1* function, indicating that wild-type SMG-1 directly or indirectly facilitates hypoxia-induced egg retention. Our screen isolate maintained expression of the *nIs470 cysl-2* transcriptional reporter, suggesting that *smg-1* is not acting upstream of HIF-1 (fig. S1, D to H).

We found that *smg-1; egl-9* double mutants laid earlier stage embryos than *egl-9* single mutants at both 20° and 25°C (Fig. 1I), indicating that loss of *smg-1* partially suppresses the *egl-9* Egl phenotype independently of *cyp-36A1* function. In addition, we found that at 25°C *smg-1* and *cyp-36A1* acted additively and thus likely function in parallel rather than in a single linear pathway (Fig. 1I).

### NMD enhances the HIF-1–mediated down-regulation of egg laying

s*mg-1* encodes a conserved kinase key in NMD, an RNA-surveillance mechanism that identifies and degrades transcripts with premature termination codons. NMD is conserved throughout eukaryotes and is critical for maintaining cellular homeostasis, particularly in response to transcript errors that can result from mutations in DNA or errors in transcriptional or splicing processes (*22*). NMD in *C. elegans* involves the functions of seven genes, *smg*-*1* to *smg-7*, each of which is necessary for the complete NMD process to occur (Fig. 2A) (*23*, *24*). Unlike in mammals (*25*), in *C. elegans*, NMD is not necessary for viability, and we observed *C. elegans smg* mutants to have few gross abnormalities compared to wild-type animals. We found that mutations in each of the seven *smg* genes suppressed the *egl-9* Egl phenotype, indicating that the process of NMD is necessary for complete HIF-1 regulation of egg laying (Fig. 2B).

**Fig. 2.**
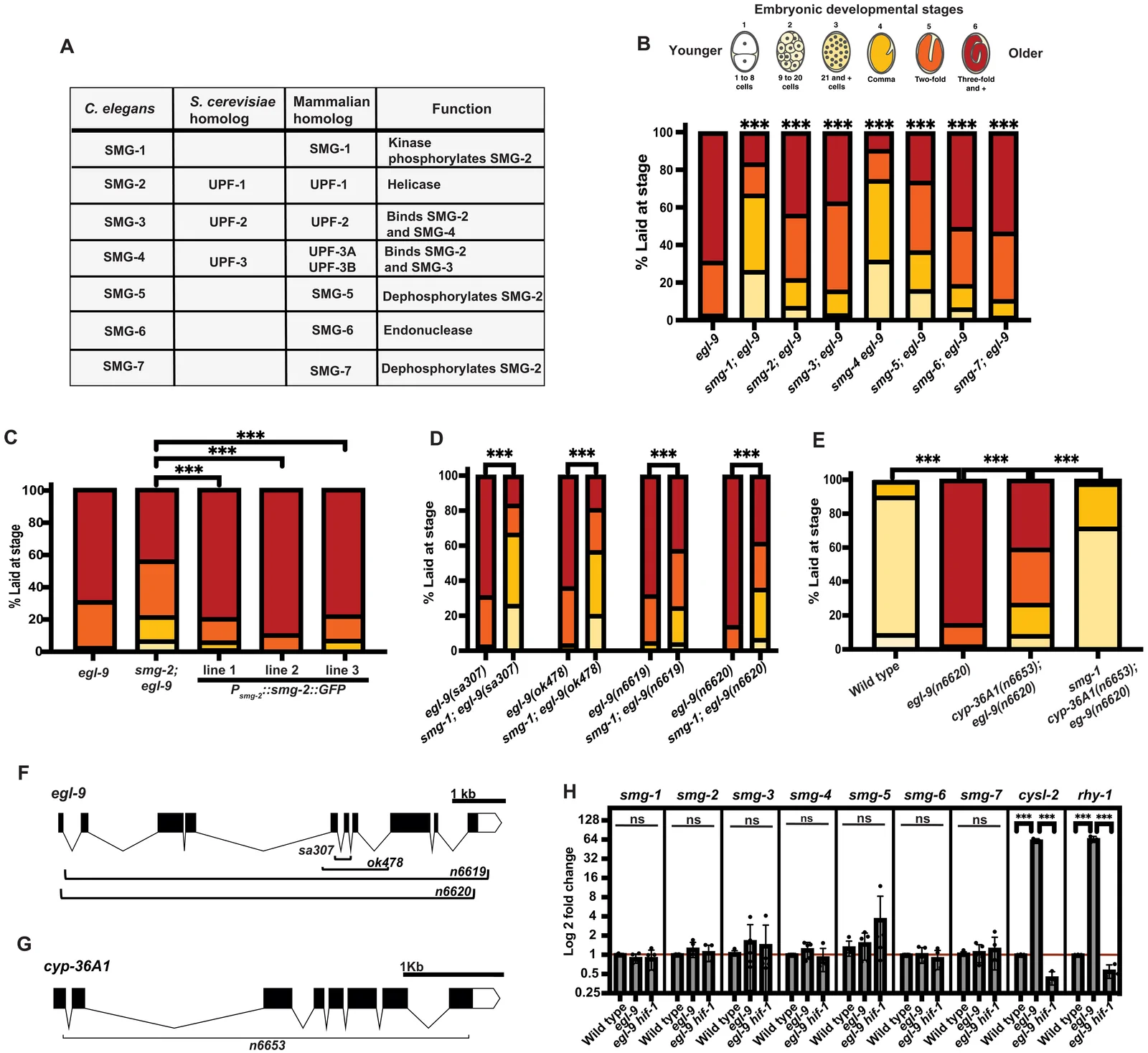
NMD enhances HIF-1–mediated down-regulation of egg laying. (**A**) Table of core NMD proteins and their known functions in *C. elegans*. (**B**) Loss of any one component of the NMD pathway inhibits the *egl-9* Egl phenotype at 20°C. Statistics show comparison to *egl-9*. Egg stages adapted from Pender and Horvitz (*18*) under CC BY 4.0 (http://creativecommons.org/licenses/by/4.0). (**C**) *smg-2* suppression of the *egl-9* Egl phenotype is completely rescued by ectopic expression of *smg-2* under its endogenous promoter. (**D**) *smg-1* suppresses multiple deletion alleles of *egl-9* at 20°C and (**E**) enhances a deletion of *cyp-36A1* at 25°C. (**F**) Schematic of the *egl-9* deletions. (**G**) Schematic of the *cyp-36A1* deletion. For all egg-laying samples, *n* > 100 embryos. (**H**) qPCR of the seven *smg* genes shows no significant changes among wild type, *egl-9*, and *egl-9 hif-1* animals. By comparison, known HIF-1 targets *cysl-2* and *rhy-1* show significant changes. Points represent the triplicate averages of independent biological replicates *n* = 2 to 5.

SMG-2 (UPF1 in yeast and mammals) is an RNA helicase core to the NMD pathway. To disrupt the NMD pathway in subsequent experiments, we used both our *smg-1(n6597)* mutation and the well-characterized *smg-2(r863)* allele, which lacks functional NMD (*26*). The suppression of *egl-9* by *smg-2* in *smg-2; egl-9* animals was completely rescued by injection of a plasmid containing *smg-2(+)* cDNA driven by the endogenous *smg-2* promoter (Fig. 2C). This result shows that the effects of *smg-2* on egg laying, and likely of other *smg* mutations, are caused by a loss of gene function. Similar rescue experiments using tissue-specific promoters (neuron, hypoderm, body-wall muscle, or intestine) indicated that expression in any of these tissues alone was not sufficient to rescue the suppression of *egl-9* by *smg-2* (fig. S2, A to E), suggesting that NMD must function in an untested tissue or in multiple tissues to coordinate the EGL-9/HIF-1 egg-laying response.

NMD mutants are often isolated as informational suppressors because of their ability to suppress nonsense alleles (*26*–*28*). In these instances, transcripts with 3′-proximal nonsense mutations undergo NMD-based degradation, resulting in a loss of gene function. However, if NMD is defective this degradation is prevented, resulting in a stabilized transcript that can generate a translational product that, despite being truncated, maintains sufficient gene function to suppress the effects of the original nonsense mutation. We found that *smg-1* was able to suppress multiple distinct *egl-9* alleles, including two deletion mutations (*n6619* and *n6620*) that each removed almost the entire open reading frame (Fig. 2, D and F). We observed the *smg-1* suppression of the *cyp-36A1; egl-9* Egl phenotype at 25°C using almost complete deletion alleles of both *cyp-36A1* and *egl-9* (Fig. 2, D to G). Since large deletion mutations cannot produce any functional translational product, these mutations cannot undergo the informational suppression that sometimes results from the loss of NMD. Together, these observations demonstrate the allele-nonspecific suppression of *egl-9* by *smg-1* and establish that NMD is not acting through regulation of *egl-9* or *cyp-36A1* mRNA as an informational suppressor.

Given the role of HIF-1 as a transcription factor, we wondered whether the NMD pathway might be transcriptionally regulated by HIF-1. To quantify mRNA levels of all seven *smg* genes in wild-type, *egl-9* (i.e., HIF-1 activated), and *egl-9 hif-1* (no HIF-1 activation) animals, we performed quantitative polymerase chain reaction (qPCR) experiments. We found no significant HIF-1–dependent changes in the expression levels of any of the seven *smg* genes, showing that *smg* transcription is not affected by HIF-1 activity (Fig. 2H).

Collectively, these data demonstrate that in *C. elegans* loss of NMD reduces HIF-1–driven egg retention and therefore that intact NMD activity is necessary for complete HIF-1–driven down-regulation of egg laying.

### Loss of NMD affects some but not all responses to HIF-1 activation

In addition to promoting egg retention, HIF-1 activation causes numerous other physiological and behavioral changes. For example, *egl-9* mutants show decreased rates of locomotion and defecation and increased resistance to various stressors, including hypoxia and oxidative stress (*18*, *29*, *30*). To determine whether NMD modulates multiple aspects of the hypoxia response, we asked if *smg-1* and *smg-2* mutations affect other HIF-1–dependent responses.

We found that the embryonic development of both *smg-1* and *smg-2* mutants was hypersensitive to hypoxia. Specifically, we placed age-matched (embryonic stage 3) recently laid embryos under hypoxic conditions (0.2% O_2_) and observed that 70% of wild-type embryos hatched into first-stage (L1) larvae within 24 hours, while the remaining 30% arrested in embryogenesis. Consistent with previous studies (*29*), we observed that this survival in hypoxia was *hif-1*–dependent, as fewer than 10% of *hif-1* embryos hatched under the same conditions. Both *smg-1* and *smg-2* mutants were significantly more hypoxia-sensitive than the wild type, with only 27 to 55% of embryos hatching (Fig. 3A). These differences were not simply reflections of baseline changes in developmental timing, as there was no significant difference in hatching rate among wild-type, *hif-1*, *smg-1*, and *smg-2* embryos grown in normoxic conditions (fig. S3A). These findings show that NMD interacts with the HIF-1 response not only in response to genetic HIF-1 activation in *egl-9* mutants but also in response to environmental hypoxia. Since *hif-1*, *smg-1*, and *smg-2* animals are non-Egl (the uterus is nonbloated and primarily stage 3 embryos are laid), the hypoxia sensitivity of these strains compared to wild type was not a consequence of differences in egg retention. These data show that loss of NMD can modulate at least two distinct aspects of the HIF-1 response, egg retention, and developmental sensitivity to hypoxia.

**Fig. 3.**
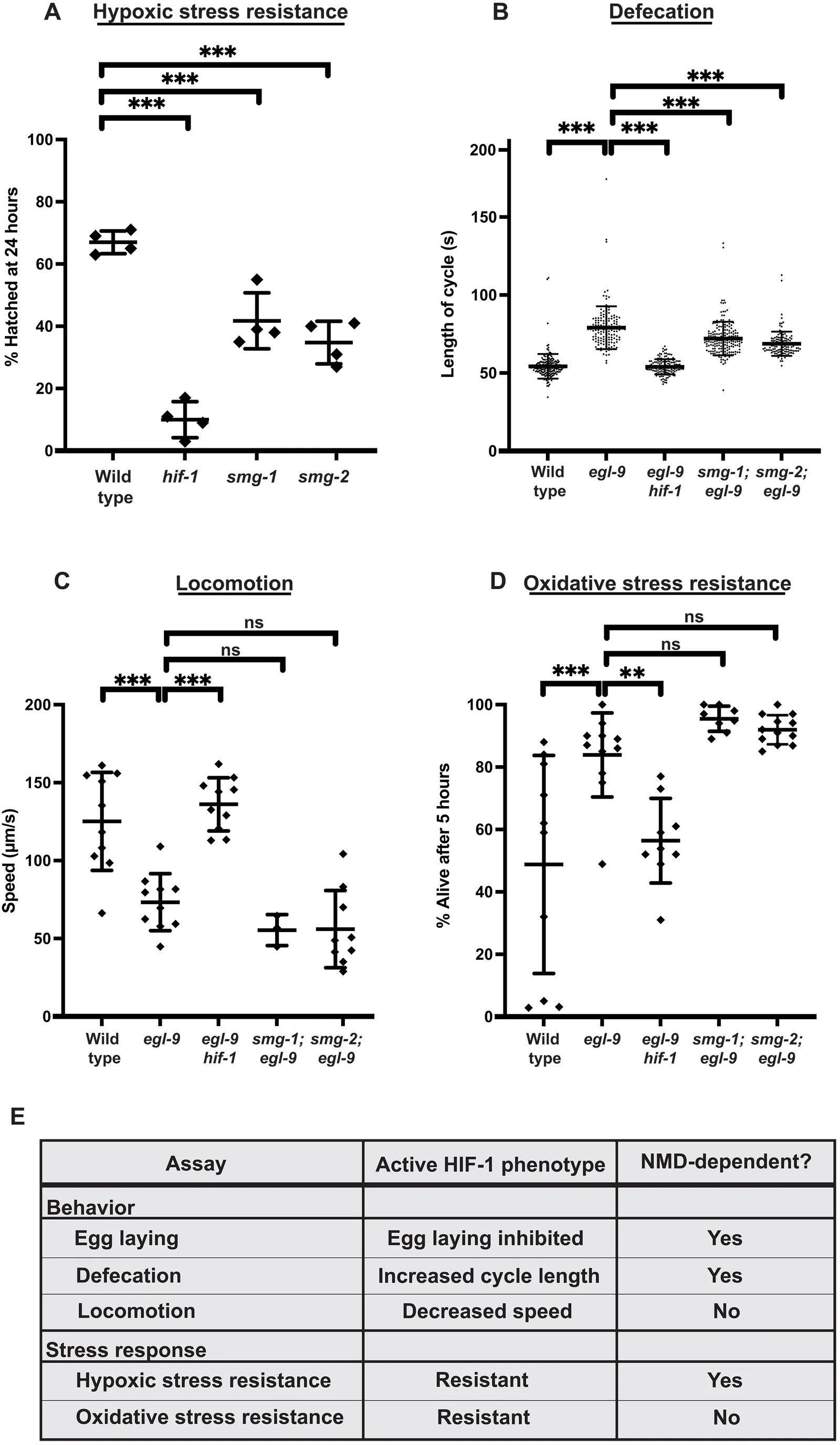
Loss of NMD modulates some, but not all, responses to HIF-1 activation. (**A**) Both *smg-1* and *smg-2* mutants are hypersensitive to hypoxia compared to wild type, showing more embryonic lethality after 24-hour exposure to 0.2% O_2_. Points show averages of 6 plates each from 4 replicate days. (**B**) Loss of *smg-1* or *smg-2* mildly suppresses the *egl-9* increase in the DMP cycle length of adults. *n* > 100 cycles, 18 to 31 animals. (**C**) Loss of NMD does not impact the HIF-1–dependent changes in locomotion. Points show plate averages. *n* = 3 to 10 plates, 10 animals per plate. (**D**) Loss of NMD does not impact *egl-9* resistance to oxidative stress, as determined by the percent of adults surviving after 5 hours on plates containing 7.5 mM t-BOOH. Points show plate averages. *n* = 8 to 11 plates, 50 animals per plate. (**E**) Summary table of all tested HIF-1–dependent phenotypes. ns, not significant.

The *C. elegans* defecation motor program (DMP) involves a coordinated cycle of stereotyped length in actively feeding wild-type animals (*31*). Consistent with previous reports (*18*), we observed that *egl-9* mutant adults had a decrease in their defecation rate, with each DMP cycle lasting 78 s, on average, compared to 54 s in wild-type adults. Similar to egg laying, this effect on defecation was *hif-1*–dependent (Fig. 3B). This phenotype was also observed for L4 animals, which have not yet begun to develop embryos, showing that the changes to the DMP were not merely a biomechanical consequence of the bloated uterus found in adults (fig. S3B). While loss of NMD alone did not affect adult defecation rates (fig. S3C), mutations in either *smg-1* or *smg-2* partially suppressed the *egl-9* DMP phenotype, with average cycle lengths of 71 and 69 s, respectively (Fig. 3B). Thus, similar to the HIF-1–mediated down-regulation of egg laying, the complete HIF-1–mediated down-regulation of defecation requires the function of the NMD pathway.

While loss of NMD affected multiple aspects of the HIF-1–driven phenotype (reduced egg laying, developmental resistance to hypoxic stress, and reduced defecation), we also identified aspects that were NMD-independent. Similar to previous studies, we observed that *egl-9* mutants had a *hif-1*–dependent decrease in locomotion rate (*18*). However, the locomotion rate of *egl-9* mutants was not affected by mutations in *smg-1* or *smg-2* (Fig. 3C). Also, previous studies reported that *egl-9* mutants display an increased resistance to oxidative stress as assayed by survival after exposure to the oxidizing agent tert-butyl hydroperoxide (t-BOOH) (*18*, *30*). We confirmed that *egl-9* mutants displayed increased resistance to t-BOOH and further observed that *egl-9 hif-1* mutants had a wild-type level of t-BOOH sensitivity, showing that this phenotype is *hif-1*–dependent. Unlike resistance to hypoxic stress, the resistance to oxidative stress by *egl-9* mutants was not affected by *smg-1* or *smg-2* mutations (Fig. 3D).

On the basis of the behavioral and stress-response assays we conducted, we conclude that NMD modulates some but not all aspects of the hypoxia response of *C. elegans* (Fig. 3E). The effects of *smg-1* and *smg-2* mutations on multiple aspects of the hypoxia response—egg laying, defecation, and hypoxic survival—indicate a substantial integration between the hypoxia and NMD pathways.

### NMD and HIF-1 act in parallel to regulate a specific subset of downstream factors

Given that both HIF-1 and the NMD machinery primarily function to adjust mRNA levels, through transcription and degradation, respectively, we hypothesized that the phenotypic differences we observed were caused by changes in mRNA levels. To analyze the effects of both HIF-1 and NMD on mRNA levels we performed whole-animal RNA sequencing (RNA-seq) studies of young adults of nine genotypes: (i) wild type, (ii) *egl-9*, (iii) *egl-9 hif-1*, (iv) *smg-1*, (v) *smg-1; egl-9*, (vi) *smg-1; egl-9 hif-1*, (vii) *smg-2*, (viii) *smg-2; egl-9*, and (ix) *smg*-2; egl-9 hif-1** (Fig. 4A). Comparisons of RNAs among these genotypes allowed us to identify transcripts that are regulated by HIF-1 and/or NMD and thereby explore the relationship between NMD and the EGL-9/HIF-1 pathway.

**Fig. 4.**
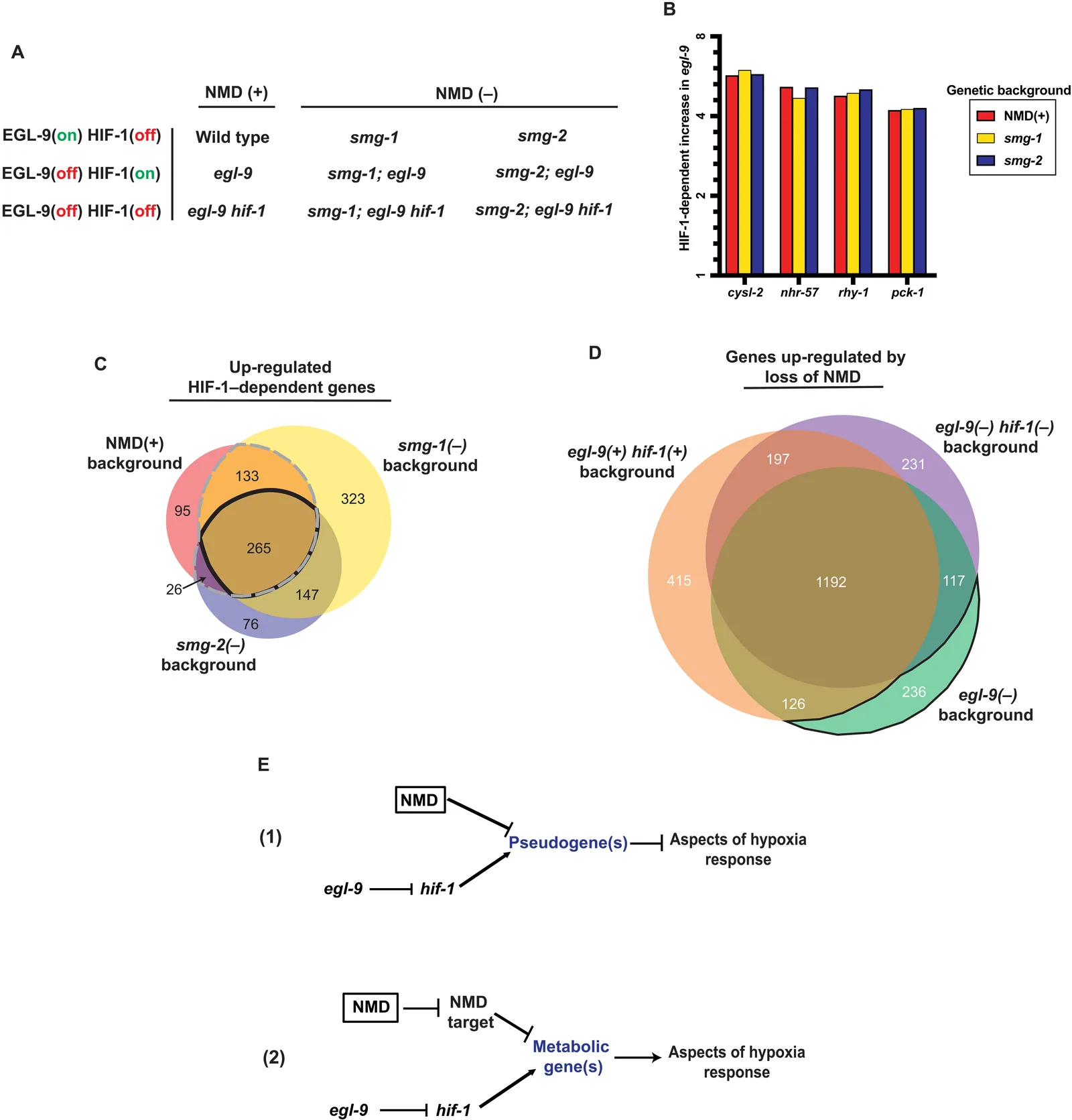
NMD and HIF-1 act in parallel to regulate a specific subset of downstream factors. (**A**) To help identify possible transcripts impacting the hypoxia response, we performed RNA-seq of nine strains with mutations in the EGL-9/HIF-1 and NMD pathways. (**B**) Fold change caused by HIF-1 activation in four well-studied HIF-1–dependent genes in the NMD(+), *smg-1(−)*, and *smg-2(−)* backgrounds show no significant decrease in HIF-1 activity. (**C**) Comparisons of genes that are significantly up-regulated in a HIF-1-dependent manner in NMD(+), *smg-1(−)*, and *smg-2(−)* backgrounds. Dashed line notes the section of genes up-regulated in NMD(+) background and at least one NMD(−) background. Solid line notes the section of genes up-regulated in all NMD backgrounds. (**D**) Comparison of genes that are up-regulated by loss of NMD in the three different hypoxia backgrounds. Solid line notes the section of genes up-regulated in *egl-9(−)* background only. (**E**) We propose two possible genetic pathways: (1) HIF-1 causes the transcription of a set of aberrant pseudogenes, which are then degraded by NMD, and (2) both NMD and HIF-1 promote the expression of a subset of metabolic genes. These two models are neither mutually exclusive nor mutually required; they may function separately or together.

To begin, we sought to confirm that loss of NMD did not cause a general decrease in HIF-1 activity, which we did using three criteria. First, we looked at four well-studied HIF-1–up-regulated genes: *cysl-2*, *nhr-57*, *rhy-1*, and *pck-1* (*32*–*34*). Corroborating our *cysl-2* reporter results (fig. S1, D to H), we found that all four genes were up-regulated by HIF-1 to similar levels in NMD(+) and both NMD(−) backgrounds (Fig. 4B). Second, expanding beyond well-studied targets, we found that most of the HIF-1–up-regulated genes (increased expression in *egl-9* versus wild type and decreased expression in *egl-9 hif-1* versus *egl-9*; fold change > 2, adjusted *P* value < 0.05) were still differentially regulated in the NMD(−) strains (Fig. 4C, fig. S4A, and data S1). Specifically, of the 519 *hif-1*–dependent up-regulated genes in the NMD(+) background, 82% were also significantly up-regulated in at least one NMD(−) background (Fig. 4C, dashed outline) and 51% in both (Fig. 4C, solid outline). We obtained similar results for HIF-1–down-regulated genes (fig. S4, B and C, and data S2). Third, we examined the magnitude of the HIF-1 up-regulation by comparing the increase in *egl-9* versus wild type in NMD(+) versus NMD(−) backgrounds. For the 519 *hif-1*–dependent up-regulated genes in the NMD(+) background, we found that 64% had similar HIF-1 response levels across NMD(+) and NMD(−) backgrounds—only 22% had a decrease in HIF-1 magnitude (*smg; egl-9* versus *smg* < 50% of *egl-9* versus wild type), and only 14% had an increase (*smg; egl-9* versus *smg* > 200% of *egl-9* versus wild type) (data S3). Collectively, these data establish that loss of NMD does not simply reduce general HIF-1 activity.

Next, we asked if HIF-1 activation causes general increases in NMD activity. While we had already observed that HIF-1 did not cause changes in *smg* transcription levels (Fig. 2H), this finding did not preclude the possibility that HIF-1 might act upstream of NMD through nontranscriptional mechanisms. If HIF-1 caused a general increase in NMD activity, then *egl-9* mutants would have increased NMD-mediated degradation, and thus NMD-target transcript levels would be decreased in *egl-9* mutants. Using a previously published list of candidate *C. elegans* NMD targets (*35*), we found that of the 581 putative NMD targets, only 3% showed significant down-regulation by HIF-1 activity in the NMD(+) background (data S4). To identify NMD-dependent transcripts in our own dataset, we compared both NMD(−) strains to the NMD(+) strain of the same *egl-9/hif-1* background, focusing on transcripts that were significantly up-regulated in both *smg-1(−)* and *smg-2(−)* backgrounds (fold change > 2, adjusted *P* value < 0.05) (Fig. 4D, fig. S4D, and data S5). Of the 2514 total transcripts that were up-regulated after loss of NMD, only 9% were exclusive to the *egl-9* background (Fig. 4D, solid outline). To determine whether HIF-1 activity affected the severity of NMD-dependent degradation, we looked at the 1192 transcripts that were up-regulated by loss of NMD in all backgrounds and found that HIF-1 activity increased the magnitude of that effect >2-fold in only 8% of transcripts (data S6). These data show that HIF-1 activity does not cause a general increase in NMD activity.

Since our data indicate that NMD does not act upstream of HIF-1 nor does HIF-1 act upstream of NMD, we proposed a genetic model in which NMD promotes aspects of the hypoxia response in parallel to HIF-1 with the two pathways coregulating a specific subset of downstream factors.

To identify specific coregulated transcripts that modulate the hypoxia response we first considered NMD targets with known downstream phenotypic impacts. The mitochondrial tyrosyl–transfer RNA (tRNA) synthetase gene *yars-2* in *C. elegans* (*36*), and multiple components of the integrated stress response (ISR) and unfolded protein response (UPR) pathways in mammals (see data S7) (*10*, *12*, *37*), have been identified as NMD targets responsible for regulating stress responses. We examined mRNA levels of genes that encode tRNA synthetases and homologs of mammalian NMD-targeted ISR and UPR pathway components. We found that none of these candidate genes displayed both NMD- and HIF-1–dependent regulation (data S7). These results suggest that the integration of NMD and the hypoxia response we observed is likely not mediated by a previously characterized pathway but instead reflects a novel mechanism.

### NMD and HIF-1 oppositely regulate a population of pseudogenes

To identify coregulated transcripts that might be responsible for controlling the hypoxia response, we first looked at transcripts that were down-regulated by NMD and up-regulated by HIF-1 activity. Specifically, we first identified transcripts that were significantly up-regulated (fold change > 2, adjusted *P* value < 0.05) in *smg-1; egl-9* and *smg-2; egl-9* double mutants compared to *egl-9* single mutants and found 1855 and 2104 genes, respectively, with 1671 genes up-regulated in both NMD(−) backgrounds (fig. S4D and data S8). We then looked at those transcripts that were also *hif-1*–up-regulated in the NMD(−) background and found 73 transcripts that were both down-regulated by NMD and up-regulated by HIF-1 activity (fig. S4E and data S8). Using WormCat (*38*), a *C. elegans* tool for identifying pathway enrichment, we found that this population of 73 transcripts had an enrichment of pseudogenes, with 16 (22%) of the coregulated transcripts being pseudogenes (data S8). On the basis of these RNA-seq data, we suggest a possible model in which HIF-1 promotes expression of a set of pseudogene transcripts that then are degraded by NMD; however, in NMD-deficient strains, levels of these otherwise-degraded pseudogene transcripts remain high after HIF-1 activation, and they then inhibit aspects of the hypoxia response (Fig. 4Ei).

### HIF-1 and NMD both up-regulate a set of metabolic genes

While our first analysis looked for coregulated genes that were up-regulated by HIF-1 and down-regulated by NMD, we also considered a model in which NMD and HIF-1 both act to up-regulate downstream factor(s). This group of genes could be both up-regulated directly by HIF-1 and, given the canonical role of NMD in degrading mRNA, up-regulated indirectly by NMD. To seek these candidate genes, we identified transcripts that were significantly down-regulated (fold change > 2, adjusted *P* value < 0.05) in *smg-1; egl-9* and *smg-2; egl-9* double mutants compared to *egl-9* single mutants and that also showed *hif-1*–dependent up-regulation. While there were more than 1000 genes up-regulated by loss of NMD, only 69 and 117 genes were down-regulated in *smg-1; egl-9* and *smg-2; egl-9*, respectively, with an overlap of 19 genes, only eight of which were also *hif-1*–up-regulated (fig. S4F and data S9). Notably, one of these genes was *cyp-36A1*, which has a known role in regulating the hypoxia response (*18*); however, a *smg-1* mutation further suppressed the *egl-9* Egl phenotype in a *cyp-36A1* complete deletion background (Fig. 2E), precluding *cyp-36A1* from being the sole NMD-dependent regulator of the *hif-1* response.

To get a better sense of possible pathways that were being affected, we relaxed our criteria to include transcripts with a fold change of >1.5. We identified 36 genes up-regulated by both NMD and HIF-1 (Fig. S4G, Data S9), and using WormCat we found that this population had a striking enrichment of metabolic genes, specifically those related to lipid and glutathione metabolism (Data S10). Based on these data, we suggest a second, not mutually exclusive, model in which both HIF-1 directly and NMD indirectly promote the expression of a set of metabolic genes that are necessary for a complete hypoxia response (Fig. 4Eii).

## DISCUSSION

We report the discovery of the integration of two fundamental and evolutionarily conserved stress response pathways, the hypoxia response pathway and the RNA-surveillance pathway of NMD. Specifically, loss of NMD reduced specific aspects of the response to HIF-1 activation (hypoxia resistance, egg retention, and decreased defecation rate), revealing that functional NMD is necessary for a complete hypoxia response. We also identified aspects of the response to HIF-1 activation (oxidative stress resistance and decreased locomotion) that were regulated independently of NMD. These findings reveal a previously unrecognized partitioning of HIF-1–dependent physiological responses.

How might NMD and the HIF-1 pathway be integrated to regulate the hypoxia response? We observed neither an NMD-dependent increase in general HIF-1 activity nor a *hif-1*–dependent increase in NMD activity, indicating that neither NMD nor HIF-1 acts strictly upstream of the other. Instead, we propose that these two conserved pathways function in parallel to coregulate one or more downstream genes and pathways. The specificity of this regulation is intriguing, as it suggests that NMD modulation of hypoxic stress is not simply caused by the additive effect of multiple cellular stressors but instead involves the specific coregulation of certain phenotypic traits and possibly specific genes. The specificity of this response might help explain why only some HIF-1–dependent responses were affected by loss of NMD; it is possible that the subset of genes coregulated by HIF-1 and NMD regulate egg laying, defecation, and hypoxic stress resistance, while oxidative stress and locomotion are controlled by other genes not affected by NMD. The specific tissues and cells in which NMD acts to modulate different aspects of the HIF-1 response are unknown and might provide insights into the specificity of NMD-dependent phenotypes.

On the basis of our RNA-seq dataset, we identified a population of 16 pseudogenes that are both up-regulated by HIF-1 activation and down-regulated by NMD. Pseudogenes frequently acquire nonsense and frameshift mutations resulting in premature termination codons that make them common targets of NMD. We suggest that these 16 pseudogenes are direct targets of NMD. We propose a model in which HIF-1 activation promotes the transcription of one or more of these pseudogenes, generating a transcript that is normally degraded by NMD but, in animals without NMD, is not degraded and instead dampens aspects of the hypoxia response (Fig. 4Ei). Eight of these pseudogenes have at least one paralog that was also HIF-1–up-regulated, suggesting that HIF-1 up-regulation of these pseudogenes might be a by-product of HIF-1 up-regulation of a related functional paralog (*39*).

A second population of mRNAs that we explored was those that are up-regulated by both NMD and HIF-1 activity, presumably directly by HIF-1 (a transcriptional activator) and indirectly by NMD (which degrades mRNAs). We identified 36 such genes, among which there was significant enrichment for metabolic genes, notably genes involved in lipid and glutathione metabolism. In response to hypoxia, cells undergo substantial metabolic reprogramming to prioritize anaerobic processes such as glycolysis, thus making *hif-1*–dependent shifts in metabolism an important aspect of the hypoxia response across cell types (*4*, *34*). We suggest a model in which the coregulated metabolic genes are components of this *hif-1*–dependent metabolic reprogramming, and in NMD mutants, undegraded NMD-target(s) inhibit the expression of one or more of these metabolic genes, resulting in decreased mRNA levels and a subsequent dampening of the hypoxia response (Fig. 4Eii).

In our models, the possible effects of NMD-targeted pseudogenes and downstream metabolic genes on the hypoxia response are neither mutually exclusive nor mutually required. Either of these two pathways could be sufficient to inhibit the hypoxia response in *smg(−)* strains, or the two might work in parallel. We also note the intriguing possibility that these two models define a single pathway in which in NMD-deficient strains the nondegraded pseudogenes described in Fig. 4Ei are in fact the NMD targets that inhibit the metabolic genes shown in Fig. 4Eii.

Variations in NMD can have profound effects on multiple aspects of human development and disease progression. For example, NMD regulates the splicing and degradation of mRNAs involved in numerous diseases, including β-thalassemia, cystic fibrosis, and cancer (*40*–*42*). NMD plays a critical role in neurodevelopment. An estimated 80% of transcripts associated with neurodevelopmental disorders (NDDs) are NMD targets (*43*), and variations in multiple components of the NMD complex have been identified as associated with NDDs. Most notably, hemizygous loss-of-function variants in UPF3B (*44*), a counterpart of the *C. elegans* protein SMG-4, are among the best established causative variants for NDDs (*45*–*47*). Hypoxia is also a critical factor for NDDs, as fetal and perinatal hypoxia exposure have been linked to increased risk of schizophrenia, ADHD, and autism spectrum disorder (ASD) (*48*), as well as to increased symptom severity among individuals with ASD (*49*). Our findings raise the intriguing possibility that humans with NMD complex variants might have increased sensitivity to hypoxic stress, thereby affecting the incidence and severity of NDDs. This impact might be regulated through increased levels of pseudogene expression and/or through decreases in metabolic pathways such as lipid or glutathione production, the latter of which has been shown to be significantly reduced in individuals with ASD (*50*).

Our discovery that NMD is necessary for the complete hypoxia response of *C. elegans* reveals a novel integration between oxygen sensing and RNA surveillance. Because the EGLN/HIF and NMD pathways both play critical roles in cellular stress responses during development and disease, this integration might have important implications for understanding how organisms respond to potentially harmful environmental perturbations in both normal and pathological settings.

## MATERIALS AND METHODS

### *C. elegans* strains and transgenes

All *C. elegans* strains were cultured as previously described (*51*). The N2 Bristol strain was used as the reference wild-type strain. The polymorphic Hawaiian strain CB4856 was used for genetic mapping (*52*). All animals were grown at 20°C unless otherwise stated. Transgenes and specific mutations used are listed below:

1) LG I: *cyp-36A1(gk824636, n6653)*, *smg-1(n6400, n6597)*, *smg-2(r863)*, and *smg-5(r860)*

2) LG III: *smg-3(r867)* and *smg-6(r896)*

3) LG IV: *nIs470[Pcysl-2::GFP*, *Pmyo-2::mCherry]*, and *smg-7(r1197)*

4) LG V: *egl-9 (sa307, ok478, n6619, n6620)*, *hif-1(ia4)*, and *smg-4(ma116)*

### Extrachromosomal arrays

The extrachromosomal arrays used in the study include *nEx3309-11[P_smg-2_::smg-2::GFP + P_ges-1_::mCherry]*, *nEx3245-6 [P_dpy-7_::smg-2::GFP + P_ges-1_::mCherry]*, *nEx3248,nEx3330 [P_hlh-1_::smg-2::GFP + P_ges-1_::mCherry]*, *nEx3249-50 [P_ges-1_::smg-2::GFP + P_ges-1_::mCherry]*, and *nEx3332-3[P_rgef-1_::smg-2::GFP + P_ges-1_::mCherry]*

### Transgenic strain construction

All *smg-2* rescue plasmids used for transgene construction were generously provided by the laboratory of S.-J. V. Lee (*36*). Transgenic strains were generated by microinjection of the germline as previously described (*53*). All *smg-2* rescue plasmids were injected at a concentration of 25 ng/μl. The *P_ges-1_::mCherry* plasmid was used as a co-injection marker and was injected at a concentration of either 5 ng/μl (*nEx3245-46* and *nEx3248-50)* or 10 ng/μl (*nEx3309-11*, *nEx3330*, and *nEx3332-3*).

### CRISPR allele construction

CRISPR alleles were generated as previously described (*54*). The *n6597* point mutation was generated using a single guide RNA (gRNA) and 100 nt ultramer (IDT) containing the desired mutation as the repair template. Deletions *n6653*, *n6619*, and *n6620* were made using multiple gRNAs throughout the gene of interest and no repair template. For all alleles, *dpy-10* was used as a co-CRISPR marker and was outcrossed to the parental strain, removing any *dpy-10* mutation.

### Mutagenesis screen for *egl-9* suppressors

To screen for suppressors of the *egl-9* Egl phenotype that functioned in parallel to *cyp-36A1*, we mutagenized *cyp-36A1(gk824636); nIs470; egl-9(sa307)* animals with EMS as described previously (*51*). Animals were grown at 25°C starting multiple generations before mutagenesis. All animals were grown and screened in normoxic conditions of ∼20% O_2_ (open laboratory air). The starting strain carried *nIs470[Pcysl-2::GFP, Pmyo-2::mCherry]*, which contains a transcriptional reporter of the HIF-1–dependent gene *cysl-2* and served as a readout of HIF-1 activity. This reporter is highly expressed in both *egl-9(lf)* single mutants and our starting strain (*19*, *20*). We used a dissecting microscope to screen the F2 progeny for suppression of the Egl phenotype, picking (i) adults that had fewer embryos in their uterus than *cyp-36A1(gk842636); egl-9(sa307)* mutants and (ii) eggs laid by the F2 animals that were at an earlier developmental stage than those laid by *cyp-36A1(gk842636); egl-9(sa307)* mutants. Screen isolates were backcrossed to determine dominant versus recessive and single-gene inheritance pattern. The screen allele *n6400*, which conferred a recessive phenotype, mapped to chromosome I based on single-nucleotide polymorphism mapping using a strain containing *cyp-36A1(gk824636); egl-9(sa307)* introgressed into the Hawaiian strain CB4856 (*52*). Whole-genome sequencing identified a mutation in *smg-1.* Regenerating this mutation with CRISPR demonstrated that it was the causative mutation, as described in the text.

### Egg-laying assay

To quantify egg-laying behavior, we scored the developmental stages of eggs laid by young adult hermaphrodites. Ten L4 animals were picked to a plate 1 day before scoring. Day 1 adults were then moved to a new plate and allowed to lay for 1 hour, after which their progeny were categorized into one of the six developmental stages previously described (*55*). Egl-D mutants, such as *egl-9*, retain eggs longer in the uterus, thus laying them at later developmental stages.

### Defecation assay

Ten animals (day-one adults or L4s) were moved to a plate seeded the previous day with 50 μl of *Escherichia coli* OP50 and they were allowed to acclimate for 10 min, after which time the plate was recorded for 10 min at a frame rate of 14 fps using a wormtracker (WormLab). Videos were analyzed, cycles of the DMP were counted, and time between each expulsion contraction was recorded (*31*). Data were not included for animals that migrated off food or out of frame for longer than 2 min.

### Locomotion assay

Ten day 1 adults were moved to a plate seeded the previous day with 400 μl of *E. coli* OP50 swirled to make a full lawn and then were allowed to acclimate for 5 min, after which time the worms were recorded for 5 min at a frame rate of 3.5 fps using a wormtracker (WormLab). Videos were then analyzed using the WormLab software to find the average speed for each animal.

### Hypoxia survival assay

The day before the assay NGM plates were seeded with 400 μl of *E. coli* OP50 swirled to cover the whole plate, and 30 to 40 L4s of each genotype were picked onto a new plate. The following day the full lawn plates were placed under a blower for 30 to 90 min to fully dry, after which 10 day 1 adults were put on each plate and allowed to lay for 1 to 2 hours before being removed from the plate. Plates were then examined and any embryos that were not developmental stage 3 (either younger or older) were removed. Plates were then placed in a hypoxic chamber (Coy Laboratory) set to 0.2% O_2_. After 24 hours, the proportion of L1s and embryos per plate was recorded.

### t-BOOH survival assay

Sensitivity to t-BOOH was assayed by placing 50 day 1 adult worms on NGM plates containing 7.5 mM t-BOOH, made using 70% t-BOOH solution (Sigma-Aldrich) and seeded with *E. coli* OP50 bacteria. Survival was evaluated after 5 hours. Animals that migrated up the side of the plate were not counted.

### RNA sample preparation

Animals were grown on NGM plates, washed off in M9, spun down at 2000 rpm for 2 min, and excess liquid was removed by aspiration. Samples were then rinsed twice with 3 ml of M9 and twice with 3 ml of ribonuclease-free water. Excess liquid was removed by aspiration, and the pellet frozen in liquid nitrogen. RLT buffer (QIAGEN) was added to the pellet, and worms were lysed using a BeadBug microtube homogenizer (Sigma-Aldrich) and 0.5-mm zirconium beads (Sigma-Aldrich). RNA was extracted using the RNeasy Mini kit (QIAGEN) according to the manufacturer’s instructions. For quantitative reverse transcription PCR (qRT-PCR) of *smg* genes mixed populations were used. For RNA-seq, 300 to 500 L4 animals were picked to a new plate and collected 6 hours later as very young adults to avoid sizable accumulation of embryos in the uterus.

### qRT-PCR protocol

Reverse transcription was performed using SuperScript IV (Invitrogen). The qPCR with SYBR green dye (Applied Biosystems) was performed by using StepOne Real Time PCR System (Applied Biosystems). Relative quantitation was performed using the ΔΔCt method. *ama-1* or *pmp-3* mRNA level was used as a control for normalization.

### Primers for qRT-PCR

The following primers were used: *ama-1* (forward: GGAGCTCGAGTGGATCTTCG; reverse: TTGTGGAGAGTCGGTTGACG), *pmp-3* (forward: ATTGCACATCCCGCATGGA; reverse: GAGGCGTTTTTGCGACCTTT), *smg-1* (forward: GGGAACCTGATAGAACAGTTTC; reverse: CTCTACATTTTTCGTAGTTTGAAG), *smg-2* (forward: GATTGCTGAGAGCCCGGAGAG; reverse: GATTGCTGAGAGCCCGGAGAG), *smg-3* (forward: CAATCAGCGATGCCTGTAGC; reverse: GCGTGACACGAGTTTGCTTT), *smg-4* (forward: CAAAACCTCCCCGTCCATCT; reverse: TTGGTGGTGACTGAAAGATCGTG), *smg-5* (forward: CGATGTTGCGCAGAAAAGGC; reverse: GATTGAACTTGTACAGTCC), *smg-6* (forward: GTCAAGGCAACGACAGAAGC; reverse: CCGACTCCTTATCGAGCACC), *smg-7* (forward: CAGTCGAAACGGATGAAGATG; reverse: GCATGAAAAGAAAAAAAACCAAAAATTATGAC), *cysl-2* (forward: GAACTTGCACTGGAGTCGGA; reverse: ATTCCGGTTCCCATGCCTTG), and *rhy-1* (forward: GCGAAGTGCGATTGTACTCATC; reverse: TAAAGTTGCATGTCGAGTCCCA).

### RNA-seq library preparation

Following RNA isolation, RNA integrity and quantity were determined by Fragment Analyzer (Agilent). Polyadenylate [poly(A)] RNA that was isolated from ∼500 ng of RNA was prepared using NEBNext poly(A) mRNA Isolation at 1/5 reaction, fragmented for 10 min, and prepared for short read sequencing using NEBNext Ultra II Directional RNA Library Prep kit for Illumina at 1/10 reaction with 12 cycles of amplification (*56*). Libraries were confirmed by fragment analysis and qPCR and sequenced with 75-nt paired end reads on an Element AVITI.

### RNA-seq data analysis

To ensure that the sequencing reads are of sufficient quality for downstream analyses, quality control (QC) was performed using an in-house pipeline developed by the MIT BioMicro Center. This pipeline includes sequencing error rate estimation, sequencing read complexity estimation, FastQC reports, sample contamination estimation, and fragment size distribution analysis. Alignment to the WBcel235 reference genome was performed using STAR/2.7.9a, and counting was conducted with RSEM/1.3.0 based on R/3.3.1. Gene-wise counts per sample were merged into a matrix, with each gene in a row and each sample in a column. This matrix serves as the foundation for differential expression analyses. Normalization and differential expression analyses were conducted using DESeq2 to assess gene expression differences across conditions. Sample hierarchical clustering and heatmap creation were performed using TIBCO Spotfire 12.4 based on Log_2_(fpkm + 1) values calculated by RSEM/1.3.0. To avoid potential noise from noncoding and nonexpressed genes, clustering and heatmap analyses were conducted using only protein-coding genes with expression.

### Statistical analysis

Mann-Whitney U tests were used to compare egg-laying data. Unpaired two-tailed *t* tests were used to compare between strains: *smg* mRNA expression as measured via qPCR, survival in hypoxia, defecation rates, locomotion rates, and survival on t-BOOH. In cases of multiple comparisons, a Bonferroni correction was applied. For all graphs: *P < 0.05, **P < 0.01, and ***P < 0.001
