# Supplementary material for: The nonsense-mediated decay RNA-surveillance pathway facilitates the hypoxia response by C. elegans
Source: Sci Adv. 2026 Oct 28;12(44):eaej4259. doi: 10.1126/sciadv.aej4259 (PMC13715696; doi:10.1126/sciadv.aej4259)
Supplement: Supplementary file 1 [file sciadv.aej4259_sm.pdf]

Science Advances
AAAS

Supplementary Materials for

The nonsense-mediated decay RNA-surveillance pathway facilitates the hypoxia response by *C. elegans*

Calista Diehl and H. Robert Horvitz

Corresponding author: H. Robert Horvitz, horvitz@mit.edu

Sci. Adv. **12**, eaej4259 (2026)
DOI: 10.1126/sciadv.aej4259

The PDF file includes:

Figs. S1 to S4
Legends for data S1 to S10

Other Supplementary Material for this manuscript includes the following:

Data S1 to S10

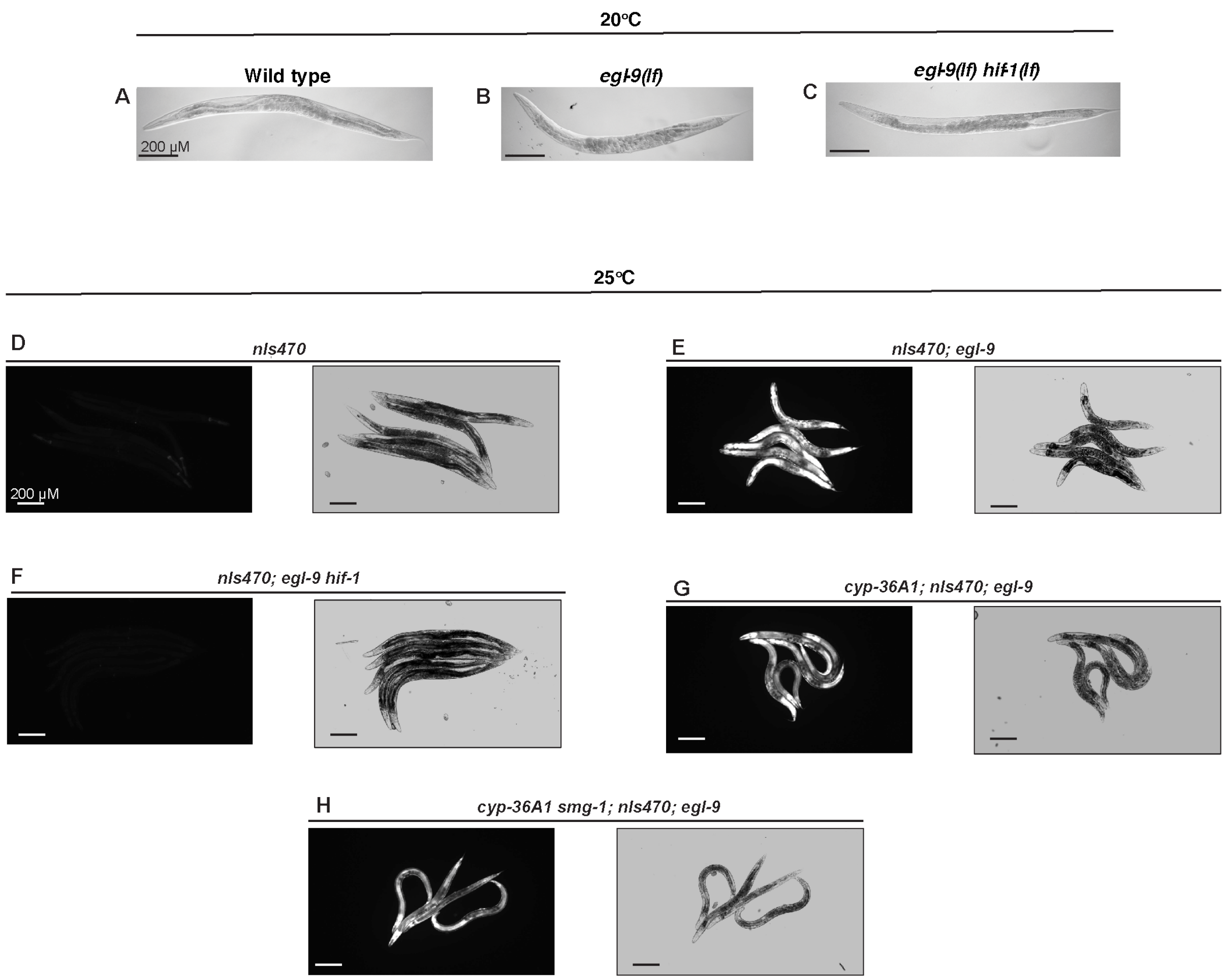

Fig. S1. Loss of *cyp-36A1* and *smg-1* do not eliminate HIF-1 activity.
Brightfield images of wild type, *egl-9* and *egl-9 hif-1* day-one adults at 20°C. (**A**) Wild type animals have a single line of embryos in their uterus, (**B**) while *egl-9* mutants have a uterus bloated with embryos. (**C**) This effect is *hif-1*-dependent and *egl-9 hif-1* double mutants have uteri similar to wild type. GFP and brightfield images of *nIs470,* a reporter of HIF-1 activity, at 25°C. Reporter expression indicates that HIF-1 activity is low in (**D**) wild type and (**F**) *egl-9 hif-1* animals, and high in (**E**) *egl-9,* (**G**) *cyp-36A1; egl-9,* and (**H**) *cyp-36A1 smg-1; egl-9* animals.

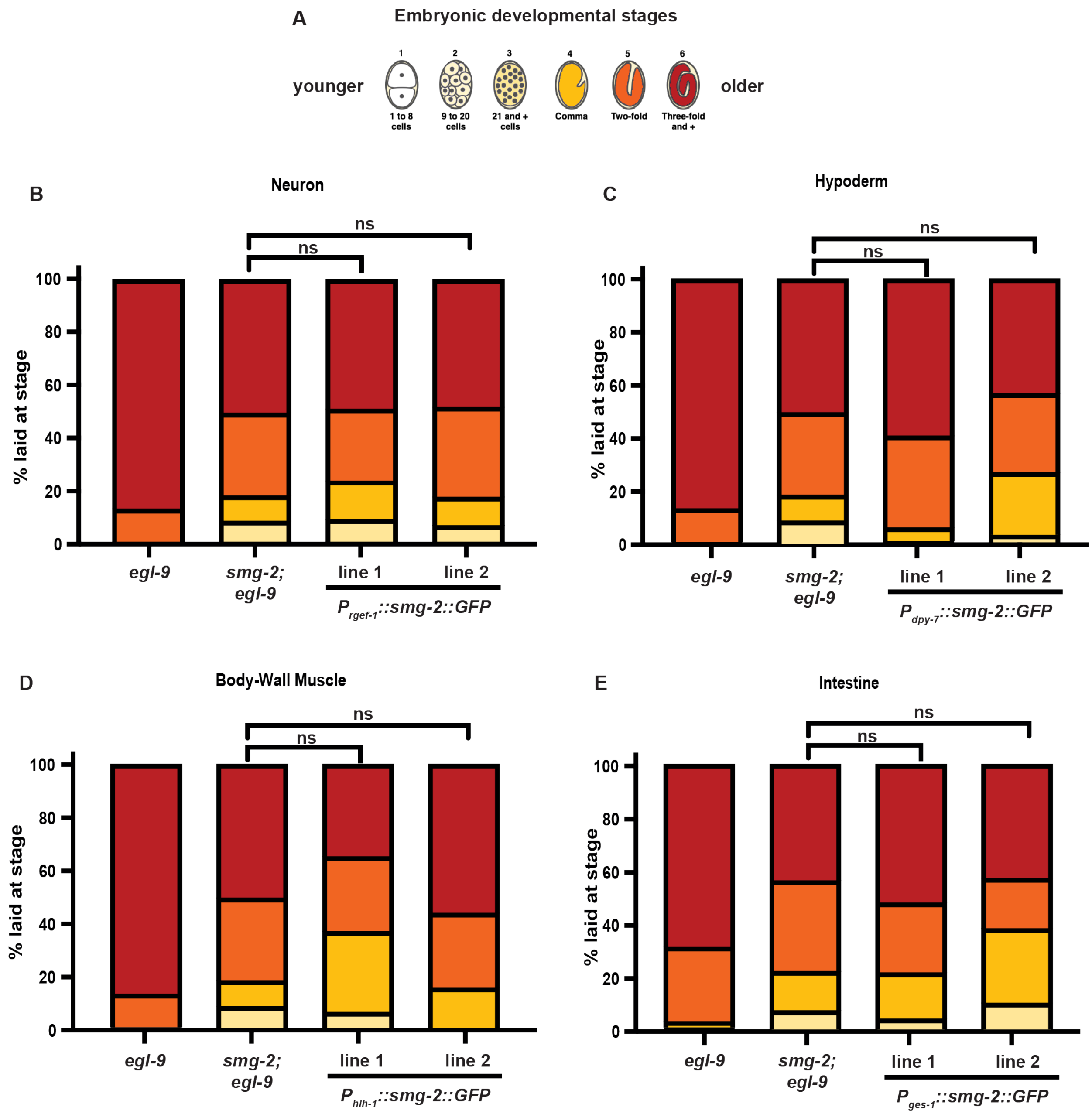

Fig. S2. Ectopic expression of *smg-2* in any single tissue is not sufficient to produce the complete hypoxia response. (**A**) The six embryonic stages used to categorize recently laid eggs. Sections of bar graphs are matched by color. Figure adapted from Pender and Horvitz *(18)*. CC BY 4.0 (http://creativecommons.org/licenses/by/4.0) Ectopic expression of *smg-2(+)* under tissue specific promoters in the (**B**) neurons, (**C**) hypoderm, (**D**) body-wall muscle, or (**E**) intestine is not sufficient to rescue the *smg-2* suppression of *egl-9*. All samples N > 50 embryos.

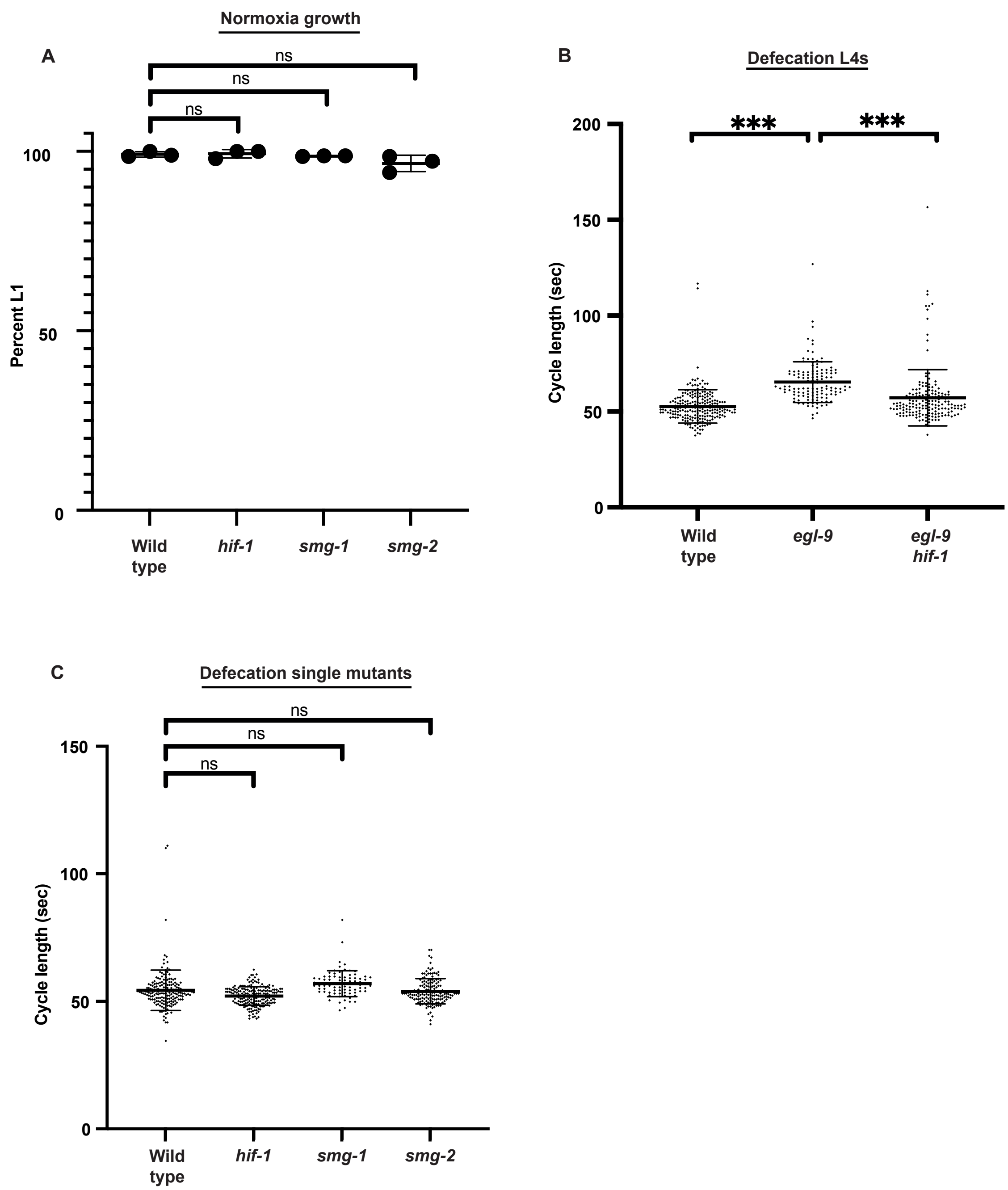

Fig. S3. Normoxic growth and defecation. (**A**) Wild-type, *hif-1, smg-1* and *smg-2* animals do not show significant changes in the proportion of embryos that reached the L1 stage after 24 hours in normoxia. Points show plate averages. N = 3 plates, 34-98 animals per plate. (**B**) *egl-9* L4s show a decrease in defecation rates similar to that of adults. Each point is one DMP cycle. N > 100 cycles, 17-22 animals. (**C**) Adult single mutants do not show significant changes in defecation. Each point is one DMP cycle. N > 50 cycles, 9-34 animals.

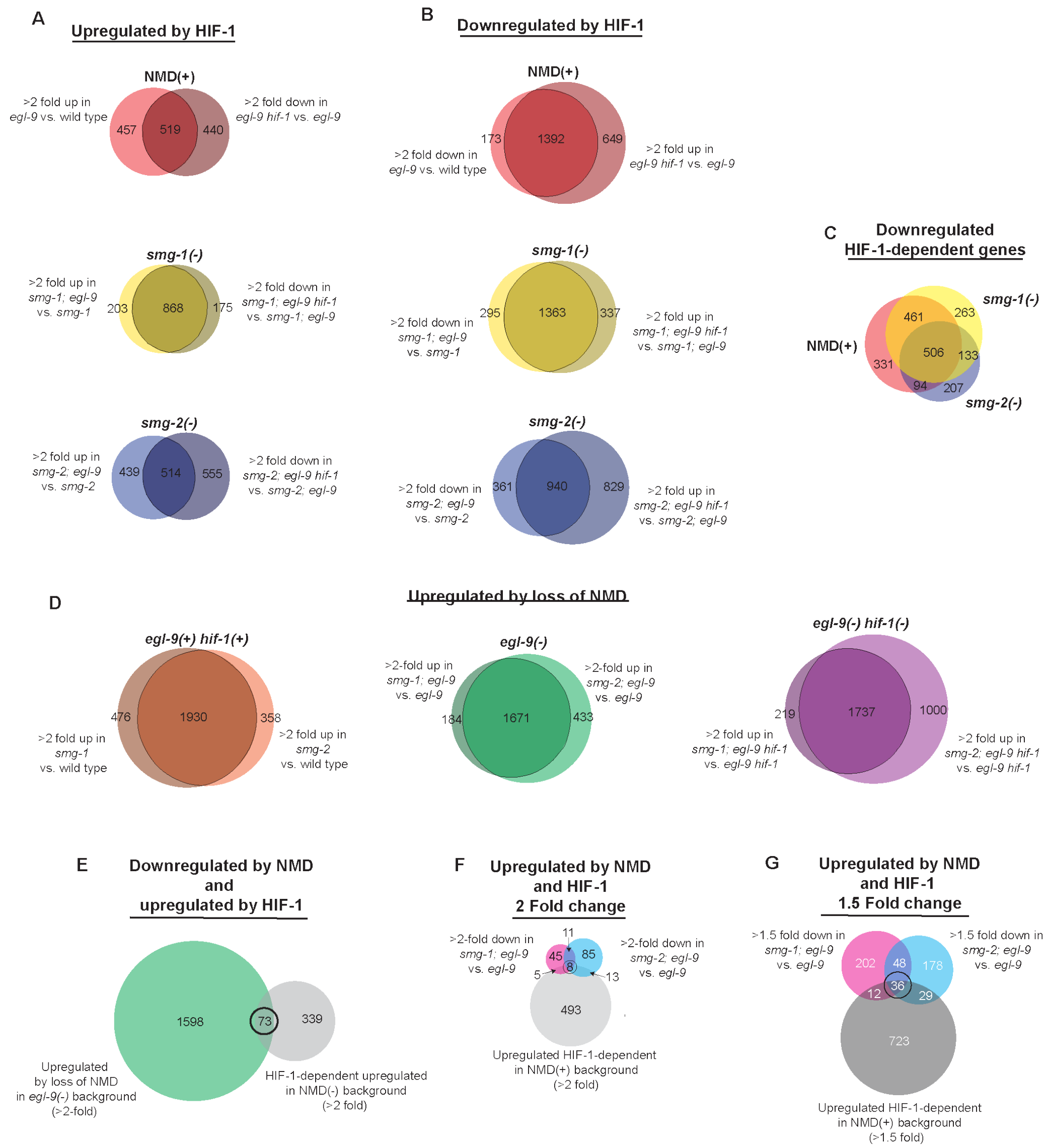

Fig. S4. HIF-1 and NMD-dependent transcriptional changes. (**A**) Genes upregulated in *egl-9* compared to wild type and downregulated in *egl-9 hif-1* compared to *egl-9* in each NMD background (NMD(+), *smg-1(-)* and *smg-2(-)*). Overlapping areas represent *hif-1*-dependent genes shown in Figure 4C. (**B**) Genes downregulated in *egl-9* compared to wild type and upregulated in *egl-9 hif-1* compared to *egl-9* in each NMD background (NMD(+), *smg-1(-)* and *smg-2(-)*). Overlapping areas represent *hif-1*-dependent genes shown in Figure S4C. (**C**) Comparisons of genes that are significantly downregulated in a *hif-1*-dependent manner in NMD(+), *smg-1(-)* and *smg-2(-)* backgrounds. (**D**) Comparison of genes that are upregulated in *smg-1* and *smg-2* vs. *smg(+)* strains in a wild type, *egl-9,* and *egl-9 hif-1* background. Overlapping regions shown in Figure 4D. (**E**) Comparison of genes that are upregulated in *smg-1; egl-9* and *smg-2; egl-9* vs. *egl-9* (middle region of Figure S4D green *egl-9(-)* Venn diagram), and genes that have *hif-1*-depenendent upregulation in the NMD(-) background (overlap of *smg-1(-)* yellow and *smg-2(-)* blue sections in Figure 4C). Comparison of genes that are downregulated in *smg-1; egl-9* and *smg-2; egl-9* vs. *egl-9* and genes that are upregulated and *hif-1*-depenendent in the NMD(+) background (NMD(+) red section of Figure 4C) based on a (**F**) >2 fold threshold and (**G**) >1.5 fold threshold.

Data S1. (separate file)

>2-Fold differentially expressed genes up-regulated by HIF-1 activation

Data S2. (separate file)

>2-Fold differentially expressed genes down-regulated by HIF-1 activation

Data S3. (separate file)

Comparison of HIF-1-dependent fold change of HIF-1-upregulated genes between NMD backgrounds.

Data S4. (separate file)

Effect of HIF-1 activation on previously published 581 candidate NMD direct targets.

Data S5. (separate file)

>2-Fold differentially expressed genes up-regulated by loss of NMD

Data S6. (separate file)

Comparison of NMD-dependent fold change of NMD-downregulated genes between *egl-9/hif-1* backgrounds.

Data S7. (separate file)

HIF-1-dependent and NMD-dependent changes in expression of 36 candidate transcripts, 32 tRNA synthetases and 4 homologs of mammalian targets.

Data S8. (separate file)

>2-fold differentially expressed genes up-regulated by HIF-1 activation or down-regulated by NMD.

Data S9. (separate file)

>1.5-fold differentially expressed genes up-regulated by HIF-1 activation or up-regulated by NMD.

Data S10. (separate file)

Functional summary of 36 genes upregulated by both HIF-1 and NMD.
